# Early Childhood Violence Exposure Patterns in The Drakenstein Child Health Study (DCHS)

**DOI:** 10.12688/wellcomeopenres.18598.2

**Published:** 2023-09-19

**Authors:** Lucinda Tsunga, Marilyn Lake, Sarah L. Halligan, Susan Malcolm-Smith, Nadia Hoffman, Jon Heron, Heather Zar, Abigail Fraser, Kirsten Donald, Dan J. Stein

**Affiliations:** 1 Paediatrics and Child Health, University of Cape Town, Cape Town, South Africa; 2 Population Health Sciences, University of Bristol, Bristol, UK; 3 Department of Psychology, University of Bath, Bath, UK; 4 ACSENT Laboratory Department of Psychology, University of Cape Town, Cape Town, South Africa; 5 Department of Psychiatry, University of Cape Town, Cape Town, South Africa; 6 The SA-MRC Unit on Child and Adolescent Health, University of Cape Town, Cape Town, South Africa; 7 Neuroscience Institute, University of Cape Town, Cape Town, South Africa

**Keywords:** childhood exposure to violence, interpersonal violence, community violence, domestic violence; polyvictimization, South Africa, preschoolers

## Abstract

**Background**: Research has highlighted high rates of exposure to violence among South African youth. However, work to date has been largely cross-sectional, focused on violence exposure during the adolescence period, and has been limited to specific types of violence exposure. We examined violence exposure in South African preschool children between 3 and 6 years of age, capturing both direct and indirect forms of violence, and tested for potential sex differences across the several types of exposures.

**Methods**: Lifetime direct and indirect exposure to domestic and community violence was measured by parental report when children were 3.5 years (N = 530), 4.5 years (N = 749) and 6 years of age (N= 417) in a South African birth cohort located in a peri-urban community.

**Results**: There are three main findings. First, a large proportion of children (72%-75%) were reported as having been exposed to some form of direct or indirect violent experience in their homes or communities from a young age. Second, there was significant polyvictimization,  with 49% of the children being exposed to more than one type of violence by age 6. Third, by 4.5 years of age, there was evidence that boys were more likely than girls to be exposed to domestic victimisation (28% vs. 17%) and polyvictimization (38% vs. 28%).

**Conclusions**: These findings highlight the high levels of violence exposure in young South African children, particularly among boys, and the need for prevention at both the community and individual levels.

## Introduction

Research conducted by the WHO indicates that homicide rates of children under the age of 5 years in South Africa (14.0 and 11.7 per 100 000 for boys and girls, respectively) were more than twice as high as the average for low and middle-income countries (LMICs; ( Krug *et al*., 2002). Moreover, the estimated economic burden of violence against children in South Africa is high. In 2015, an estimated 2.3 million disability-adjusted life years (DALYs) were lost in South Africa due to non-fatal violence against children, and 84,287 due to fatal child-focused violence. The estimated economic cost of DALYs lost to violence against children in 2015 was ZAR173 billion (USD 13.5 billion)—or 4.3% of the country’s gross domestic product that year ( Fang *et al*., 2017).

The causes of violence in South Africa are multifaceted. It is considered to be embedded in the colonial history and legacy of apartheid as under the apartheid government violence was widely accepted and normalised ( Bruce, 2009; Ward *et al*., 2013). Extensive poverty, inequality, high unemployment rates together with a fragile law enforcement system, the rise of urbanisation, poor housing and education outcomes all play a role in the perpetuation of community violence ( Bruce *et al*., 2007; Seedat *et al*., 2009). Furthermore, intimate partner violence (IPV) and physical disciplining methods such as corporal punishment are widely tolerated and accepted as social norms and are intergenerationally transferred. For example, in one study, 58% of South African caregivers reported having smacked their children at least once and 33% reported using an object such as a belt ( Dawes *et al*., 2005). A cycle of vulnerability to violence may also exist, with maltreatment among mothers in childhood being linked with an increased risk of experiencing IPV in pregnancy, and adulthood ( Barnett *et al*., 2018). Sex differences in violence exposure types have also been reported, where teenage boys have a higher risk of becoming victims of homicide and community violence than girls ( Mathews *et al*., 2013).

Despite compelling evidence of substantial violence exposure among South African youth, and in other LMIC populations, substantial gaps exist in our knowledge. First, existing studies have focused on adolescents rather than younger children, with little evidence on preschool children in particular. Second, most work has been cross-sectional, with few longitudinal studies. Understanding how patterns of violence exposure may change with age is relevant to developing targeted prevention strategies. Third, studies have often focused on single forms of violence exposure (e.g., IPV), and there is a need to explore a range of direct and indirect forms of trauma exposure to provide a full picture of the risks to children. Relatedly, there is limited research on polyvictimisation - the phenomenon where individuals are exposed to multiple forms of trauma - despite evidence that polyvictimisation is a particular risk factor for poor child outcomes ( De Bellis *et al*., 2013; Finkelhor *et al*., 2015; Ford, 2021; Haahr-Pedersen *et al*., 2020a; Kaminer *et al*., 2013a; Le *et al*., 2018). Finally, little work has described sex differences in exposure to violence, which may be important to consider in the development of targeted intervention strategies. In sum, research investigating the exact patterns of violence exposure in the day-to-day lives of preschool children is needed. This is essential in South Africa where interpersonal violence is particularly high.

We addressed the above research gaps using a longitudinal South African birth cohort, the Drakenstein Child Health Study (DCHS), which provides a unique resource for doing so. Previous studies using the DCHS cohort found high levels of IPV and childhood trauma among mothers ( Stein *et al*., 2015). Here we explored the violence exposure patterns in the DCHS children born to these mothers, who consisted of males and females aged between 3 to 6 years, and where both direct and indirect forms of violence were longitudinally measured across 3 different time points. Sex differences in exposure patterns were also examined as well as the extent of polyvictimisation in this young age group.

## Research Methods

### Study Design

The DCHS is a longitudinal study employing a multidisciplinary approach to investigate the early-life determinants of child health in two peri-urban communities in the Drakenstein sub-district of the Cape Winelands, Western Cape, South Africa ( Zar *et al*., 2015). Longitudinal measurements of risk factors in seven domains (environmental, infectious, nutritional, genetic, psychosocial, maternal and immunological) are used to investigate child health in addition to maternal and paternal health. The early-life component of the study centres on a wide range of developmental outcomes in domains that include physical health and growth as well as neurodevelopmental, cognitive and psychological health ( Donald *et al*., 2018).

### Study Setting

The study population is characterised by low-socioeconomic status (SES) and multiple psychosocial risk factors are prevalent, such as single-parent households, high rates of psychological distress and violence exposure, HIV and illicit drug use, high levels of violence and intimate partner violence ( Groves *et al*., 2015) and low levels of employment and educational achievement ( Stein *et al*., 2015). The population is a stable one, with low immigration or emigration and over 90% of the inhabitants use the public health care systems. In view of the factors above, the DCHS cohort can be considered representative of other South African and LMICs peri-urban communities.

### Participants

Pregnant women were recruited whilst attending one of the two primary healthcare clinics in the area, Mbekweni (serving a predominantly Black African ancestry community) and TC Newman (serving a predominantly mixed ancestry community ^1^) between March 2012 and March 2015. Pregnant women were enrolled if they were at least 18 years of age, received their antenatal care at either of the two clinics and planned on remaining in the area for at least one year. Mothers who consented were enrolled at 20–28 weeks’ gestation and mother-child dyads have been followed longitudinally; to date, the oldest children are 10 years of age. At enrolment, mothers provided informed written consent and were further re-consented annually after childbirth. Mother-child dyads attended follow-up visits at the two clinics and Paarl Hospital ( Stein *et al*., 2015). Trained study staff from the community guided the informed consent process with the mothers in the mothers’ language of choice, isiXhosa, Afrikaans or English.

**Figure 1.**
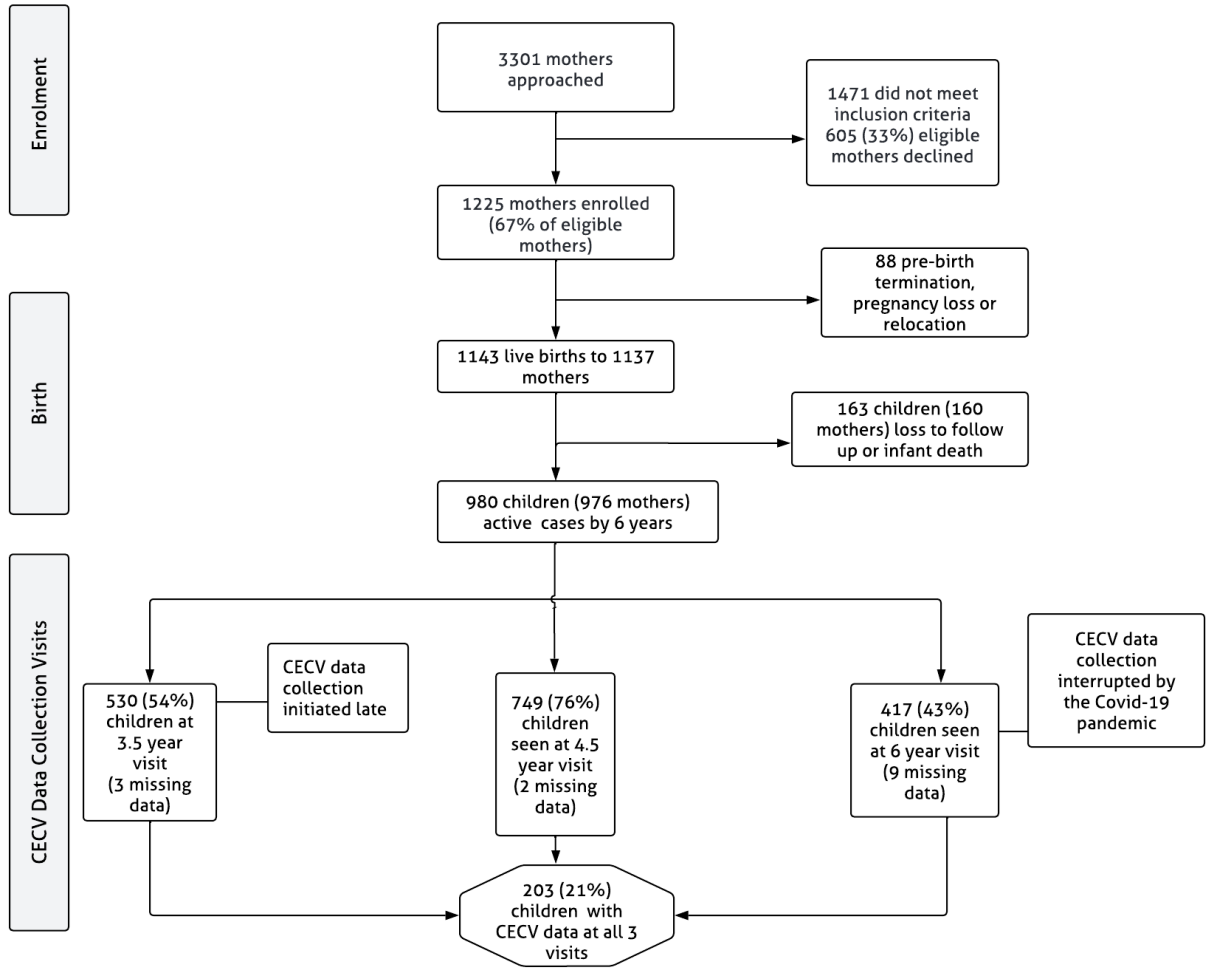
DCHS flowchart for CECV data collection.

A total of 1137 mother-child dyads were enrolled in the study, from these, four mothers had twins and one had triplets. Therefore, the total number of children who were enrolled in the study is 1143. Due to attrition (see Figure 1 for details), the current sample in the DCHS is 980. All the children were born at the main hospital and child sex was established at birth. Here, sex, classified as female or male, refers to a set of biological attributes in humans linked with physical and physiological features including chromosomes, gene expression, hormone function and reproductive/sexual anatomy ( Coen & Banister, 2012).

### Procedures and Data Collection Measures

***Sociodemographic characteristics.*** A questionnaire adapted from the South African Stress and Health (SASH) Study ( Myer *et al*., 2008) was used to collect data on sociodemographic variables such as household income, employment status and education. The questionnaire was administered by trained study staff in interview format antenatally at 28 to 32 weeks’ gestation and during annual study visits.

***Violence Exposure.*** The Child Exposure to Community Violence Checklist (CECV) is a parent-report measure comprised of 35 items assessing the children’s lifetime exposure to domestic, school and community violence.

A standard forward and backward approach ( Smit *et al*., 2006) was used to translate the measure from English to isiXhosa and Afrikaans. Translations were further cross-checked with study staff based in study communities to ensure that the appropriate dialect was used. The measure was administered by trained research assistants in Afrikaans for the Afrikaans-speaking participants and isiXhosa through the aid of interpreters for the isiXhosa-speaking participants. An interview format was adopted due to the low levels of literacy in the sample, such that mothers were afforded the means to request for clarification when needed.

The CECV version used in the study was adapted to more correctly target the South African population ( Bruwer *et al*., 2008; Fincham *et al*., 2009) as well as the study focus. It has shown good psychometric properties such as good internal reliability in previous South African studies such as *r* = .93 ( Fincham *et al*., 2009), *r* =.86 ( Kaminer *et al*., 2013d) and *r* = .85 - .87 ( Kaminer *et al*., 2013b). The checklist is coded on a four-point Likert scale rating the frequency of exposure ranging from “0” (never), to “3” (many times). Here we collapsed the responses to create a binary yes/no indicator of exposure for each item by combining exposure to violence ratings “once”, “a few times” and “many times” to indicate “yes” to violence exposure and retained the rating “never” as an indication of no exposure. This was done to enable us to create subscales and a total exposure score, that clearly reflect the number of types of violence exposures, as opposed to conflating exposure frequency and type.

The total exposure score ( *Overall Violence Exposure, α* = 0.88) was generated from the CECV by summing over the 35 items with high scores indicating a greater frequency of exposure (range 0–35). We also created four subscales from the CECV items to characterise violence exposure patterns in this cohort, namely, *Witnessing Community Violence* (10 items, α = .72), *Community Victimisation* (8 items, α = .75), *Witnessing Domestic Violence* (6 items, α = .75) and *Domestic Victimisation* (11 items, α = .79) consistent with previous studies that used this measure ( Kaminer *et al*., 2013c; Kaminer *et al*., 2013d). We defined polyvictimisation as a score of 1+ on two or more subscales.

### Ethical considerations

All procedures performed were in accordance with the ethical standards of the Medical Research Council in South Africa and with the Helsinki Declaration (2013. The study protocol including consent forms was approved by the Faculty of Health Sciences Research Ethics Committee, University of Cape Town (401/2009), and by the Western Cape Provincial Research committee (2011RP45). Given the content of the CECV scale, a key obligation in the study was to flag instances of abuse, trauma and mental health issues. An active referral system was in place for both mothers and children supported by close relationships between study staff and provincial health staff. Furthermore, all women participating in the study, regardless of specific mental or physical health problems, were informed about social and support service providers available to them.

### Data analysis

Data analysis was performed using R Statistical Software (version 4.0.2) and R Studio (version 1.3.1073) for Mac. Given that the exact timing of violence exposure was not measured, each timepoint was cross-sectionally analysed. Prevalence of *Overall violence Exposure* and subtypes of violence were descriptively summarised using counts and proportions. We examined the distributions of subscales and *Overall violence Exposure* using the Shapiro-Wilk test of normality. The frequency of polyvictimisation was also explored. Chi-square analyses were conducted to explore sex and sociodemographic differences in violence exposure.

### Missing Data 

Item nonresponse occurred on the CECV scale in some of the cases where the CECV was completed at each visit. At the 3.5-year visit (N = 530), 3 cases (0.6%) had incomplete data, at the 4.5-year visit (N = 749), 2 cases (0.3%) had incomplete data and at the 6-year visit (N = 417), 9 cases (2.2%) had incomplete data ( Figure 1). 

In these cases, item nonresponse was handled by imputing missing values using the single modal imputation method for each measurement occasion separately. Modal imputation was carried out using Base R functions of R Statistical Software (version 4.0.2)

## Results

The sample sizes at each age/visit differ from the current total DCHS sample ( *N* = 980). This is due to late CECV data collection initiation at the first study visit (age 3.5), data collection interruptions at the 6-year visit due to the COVID-19 pandemic, and non-response at any one of the time points. Only 203 children had CECV data at all 3 visits ( Figure 1). Participants’ sociodemographic characteristics are summarized in Table 1. Most of the households are from a low SES, with 49% having a monthly household income between 1000–5000 ZAR (62 - 310 USD), and 38% earning less than 1000 ZAR (62 USD) per month. Many children came from single-parent households and lived with on average 4 to 5 people. Furthermore, mothers reported high levels of unemployment and low levels of educational attainment. The distribution of characteristics was similar in participants who attended the 3.5, 4.5- and 6-year clinics, the 203 children with CECV data at all 3 visits and the cohort overall.

**Table 1.** Demographic profiles and baseline descriptive statistics of the sample stratified by visit.

	3.5 years ( *N* = 530)	4.5 years ( *N* = 749)	6 years ( *N* = 417)	Subsample (N=203)	Full cohort ( *N* = 1137)
**Sex**					
Female	262 (49%)	371 (50%)	198 (48%)	95 (47%)	550 (48%)
**Average household income per month**					
<1000 ZAR (62 USD)	179 (34%)	272 (36%)	162 (39%)	76 (37%)	430 (38%)
1000–5000 ZAR (62 – 310 USD)	282 (53%)	388 (52%)	199 (48%)	97 (48%)	553 (49%)
>5000 ZAR (310 USD)	69 (13%)	89 (12%)	56 (13%)	30 (15%)	154 (14%)
**Mother's Education**					
Primary	38 (7%)	60 (8%)	42 (10%)	8 (9%)	86 (8%)
Some Secondary	292 (55%)	411 (55%)	213 (51%)	100 (49%)	606 (53%)
Completed Secondary	171 (32%)	235 (31%)	146 (35%)	75 (37%)	372 (33%)
Any tertiary	29 (6%)	43 (6%)	16 (4%)	10 (5%)	73 (6%)
**Mother’s Employment Status**					
Unemployed	388 (73%)	560 (75%)	313 (75%)	149 (73%)	831(73%)
**Mother’s Partnership status**					
Married/cohabiting	223 (42%)	301 (40%)	164 (39%)	77 (38%)	458 (40%)
**Number of people in the household**					
Median (IQR)	4(3–6)	5 (3–6)	4 (3–6)	4(3–6)	4 (3–6)

## Violence exposure patterns

The proportions of children exposed to any form of violence, as well as subscale scores and item level exposure by age at measurement are presented in Table 2. Exposure to any form of violence by each of the visits was 72%, 75% and 76%, at ages 3.5, 4.5 and 6 years respectively. *Witnessing Community Violence* was the most prevalent trauma: (62%, 67% and 69%), followed by *Domestic Victimisation* (24%, 23% and 31%), *Witnessing Domestic Violence* (28%, 24% and 21%) and *Community Victimisation* (9%, 9% and 14%). Similar prevalence rates are reported in the subsample (N = 203) of those participants consistently seen at all visits, see Supplementary Table 1.

**Table 2.** Prevalence of Exposure to Specific Violence Types at Each Study Visit.

	3.5 years ( *N* = 530)	4.5 years ( *N* = 749)	6 years ( *N* = 417)
	Exposure at Least Once		
**Exposure to any form of violence**	**383 (72.3%)**	**562 (75.0%)**	**318 (76.3%)**
***Witnessing community violence***	***329 (62.1%)***	***499 (66.6%)***	***287 (68.8%)***
Heard gunshots	199 (37.5%)	315 (42.1%)	201 (48.2%)
Seen someone beaten up in the neighbourhood	219 (41.3%)	341 (45.5%)	184 (44.1%)
Seen dead body in the neighbourhood	35 (6.6%)	64 (8.5%)	39 (9.4%)
Seen somebody point a gun at another in the neighbourhood	31 (5.8%)	42 (5.6%)	34 (8.2%)
Seen somebody get shot in the neighbourhood	8 (1.5%)	22 (2.9%)	22 (5.3%)
Seen somebody point a knife at another in the neighbourhood	75 (14.2%)	119 (15.9%)	72 (17.3%)
Seen somebody get stabbed in the neighbourhood	47 (8.9%)	65 (8.7%)	50 (12.0%)
Seen someone forced to do something sexual neighbourhood	3 (0.6%)	1 (0.1%)	7 (1.7%)
Child known someone killed by another	15 (2.8%)	30 (4.0%)	22 (5.3%)
Seen someone being killed by another person elsewhere	7 (1.3%)	11 (1.5%)	9 (2.2%)
***Community victimisation***	***45 (8.5%)***	***68 (9.1%)***	***58 (13.9%)***
House robbery child present	24 (4.5%)	27 (3.6%)	34 (8.2%)
Someone threatened to beat up the child at school or creche	12 (2.3%)	14 (1.9%)	22 (5.3%)
Someone threatened to beat up the child elsewhere	12 (2.3%)	15 (2.0%)	13 (3.1%)
Child been beaten up elsewhere	7 (1.3%)	22 (2.9%)	13 (3.1%)
Someone elsewhere threatened to kill the child	1 (0.2%)	3 (0.4%)	4 (1.0%)
Someone at school or creche threatened to shoot or stab the child	2 (0.4%)	1 (0.1%)	7 (1.7%)
Someone elsewhere threatened to shoot or stab the child	2 (0.4%)	1 (0.1%)	5 (1.2%)
Someone shot or stabbed the child elsewhere	2 (0.4%)	1 (0.1%)	4 (1.0%)
***Witnessing domestic violence***	***147 (27.7%)***	***179 (23.9%)***	***86 (20.6%)***
Seen grownups at home hit each other	138 (26.0%)	169 (22.6%)	73 (17.5%)
Seen somebody point gun at another at home	10 (1.9%)	5 (0.7%)	10 (2.4%)
Seen someone at home get stabbed	21 (4.0%)	22 (2.9%)	22 (5.3%)
Seen someone at home get shot	3 (0.6%)	2 (0.3%)	5 (1.2%)
Seen someone forced to do something sexual	2 (0.4%)	2 (0.3%)	9 (2.2%)
Seen someone being killed by another person at home	2 (0.4%)	7 (0.9%)	6 (1.4%)
***Domestic victimisation***	***125 (23.6%)***	***169 (22.6%)***	***130 (31.2%)***
Someone threatened to beat up the child at home	12 (2.3%)	15 (2.0%)	25 (6.0%)
Child been beaten up at home	12 (2.3%)	16 (2.1%)	15 (3.6%)
Someone at home threatened to kill the child	4 (0.8%)	3 (0.4%)	8 (1.9%)
Family member threatened to shoot or stab the child	1 (0.2%)	1 (0.1%)	5 (1.2%)
Someone shot or stabbed the child at home	2 (0.4%)	3 (0.4%)	4 (1.0%)
Someone made the child do something sexual	5 (0.9%)	6 (0.8%)	10 (2.4%)
Family member shouts at the child fiercely and loudly	60 (11.3%)	60 (8.0%)	39 (9.4%)
Anyone at home used a stick or belt or hard item to hit the child	34 (6.4%)	50 (6.7%)	54 (12.9%)
Anyone at home hit the child so hard they were hurt	19 (3.6%)	26 (3.5%)	23 (5.5%)
Anyone at home said the child would be sent away or kicked out	12 (2.3%)	17 (2.3%)	31 (7.4%)
Anyone at home called the child horrible names	43 (8.1%)	66 (8.8%)	53 (12.7%)

Looking at all three time points, the most prevalent exposures in the *Witnessing Community Violence* subscale were *hearing gunshots* (38% – 48%) and *seeing someone beaten up in the neighbourhood* (41% – 46%). In the *Community Victimisation* subscale, the most common exposure was *House robbery occurring whilst the child was present* (4% – 8%). *Seeing grownups fighting* (18% – 26%) was the most common exposure in the *Witnessing Domestic Violence* subscale. The most prevalent *Domestic Victimisation* subtypes were the *child being hit by a stick*, *belt or another hard item at home* (6% – 13%), the *child being shouted at fiercely and loudly by a family member* (8% – 11%) and *child being called horrible names by someone at home* (8% – 13%).

Table 3 provides summary statistics (proportion exposed, median, IQR and range) for the overall violence score and subscales by age and sex. The only evidence for sex differences was seen for reported exposure to *Domestic Victimisation* by age 4.5, where more boys than girls had reports of exposure (28% vs. 17%). 

**Table 3.** Prevalence of forms of violence by each visit stratified by sex. *p-value*: Chi-square test.

	3.5 Years ( *N* = 530)			4.5 Years ( *N* = 749)			6 Years ( *N* = 417)		
	Female ( *n =* 262)	Male ( *n* = 268)	*p-value*	Female ( *n* = 371)	Male ( *n* = 378)	*p-value*	Female ( *n* = 198)	Male ( *n* = 219)	*p-value*
Overall Violence Exposure									
Exposed	179 (68%)	203 (77%)	0.07	273 (74%)	288 (76%)	0.46	148 (75%)	164 (75%)	1.00
Min / Max	0 / 35	0 / 15		0 / 14	0 / 35		0 / 34	0 / 35	
Med (IQR)	1 (0;3)	2 (1;3)		1 (0;2)	2(1;3)		1(1;3)	2(1;4)	
Witnessing Community Violence									
Exposed	153 (58%)	175 (65%)	0.12	237 (64%)	261 (69%)	0.16	129 (65%)	154 (70%)	0.31
Min / Max	0 / 10	0 / 8.0		0 / 6	0 / 10		0 / 9	0 / 10	
Med (IQR)	1 (0;2)	1.0 (0;2)		1(0;2)	1 (0;2)		1 (0;2)	1 (0;3)	
Community Victimisation									
Exposed	19 (7%)	24 (9%)	0.58	28 (8%)	39 (10%)	0.23	19 (10%)	32 (15%)	0.16
Min / Max	0 / 8	0 / 3.0		0 / 2	0 / 8		0 / 8	0 / 8	
Med (IQR)	0 (0;0)	0 (0;0)		0 (0;0)	0 (0;0)		0 (0;0)	0 (0;0)	
Witnessing Domestic Violence									
Exposed	66 (25%)	80 (30%)	0.27	82 (22%)	96 (25%)	0.33	39 (20%)	41 (19%)	0.90
Min / Max	0 / 6	0 / 4		0 / 4	0 / 6		0 / 6	0 / 6	
Med (IQR)	0 (0;1)	0 (0;1)		0 (0;0)	0 (0;1)		0 (0;0)	0 (0;0)	
Domestic Victimisation									
Exposed	54 (21%)	69 (26%)	0.19	64 (17%)	104 (28%)	0.001	51 (26%)	73 (33%)	0.11
Min / Max	0 / 11	0 / 6		0 / 5	0 / 11		0 / 11	0 / 11	
Med (IQR)	0 (0;0)	0 (0;1)		0 (0;0)	0 (0;1)		0 (0;1)	0 (0;1)	

Table 4 provides frequencies of violence exposure by marital status, household income and age. There was no evidence of associations between violence exposure and maternal marital status or social economic status (indicated by household income). 

**Table 4.** Violence Exposure by Marital Status and Household Income. *p-value*: Chi-square test.

	Overall Viole *n*ce Exposure		Witnessing Community Violence		Community Victimization		Witnessing Domestic Violence		Domestic Victimization	
3.5 years ( *N* = 530)										
	Exposed ( *n* = 382)	*p-value*	Exposed ( *n* = 328)	*p-value*	Exposed ( *n* = 43)	*p-value*	Exposed ( *n* = 146)	*p-value*	Exposed ( *n* = 123)	*p-value*
**Mother's Marital Status**										
Married/cohabiting (n = 223)	170 (76%)	0.09	143 (64%)	0.41	22 (10%)	0.27	65 (29%)	0.55	54 (24%)	0.72
Single (n = 307)	212 (69%)		185 (60%)		21 (7%)		81 (26%)		69 (22%)	
**Household Income**										
< R1000/m (n = 179)	133 (74%)	0.59	121 (68%)	0.15	10 (6%)	0.15	46 (26%)	0.62	40 (22%)	0.54
R1000–5000/m (n = 282)	198 (70%)		167 (59%)		24 (9%)		78 (28%)		70 (25%)	
>R5000/m (n = 69)	51 (74%)		40 (58%)		9 (13%)		22 (32%)		13 (19%)	
4.5 years ( *N* = 749)										
	Exposed (n = 561)		Exposed (n = 498)		Exposed (n = 67)		Exposed (n = 178)		Exposed (n = 168)	
**Mother's Marital Status**										
Married/ cohabiting (n = 301)	230 (76%)	0.49	202 (67%)	0.83	23 (8%)	0.37	78 (26%)	0.30	61 (20%)	0.28
Single (n = 448)	331 (74%)		296 (66%)		44 (10%)		100 (22%)		107 (24%)	
**Household Income**										
< R1000/m (n = 272)	207 (76%)	0.82	185 (68%)	0.65	26 (10%)	0.50	58 (21%)	0.49	64 (24%)	0.09
R1000–5000/m (n = 388)	287 (74%)		252 (65%)		36 (9%)		97 (25%)		77 (20%)	
>R5000/m (n = 89)	67 (75%)		61 (69%)		5 (6%)		23 (26%)		27 (30%)	
6 years ( *N* = 417)										
	Exposed (n = 312)		Exposed (n = 283)		Exposed (n = 51)		Exposed (n = 80)		Exposed (n = 124)	
**Mother's Marital Status**										
Married/ cohabiting (n = 164)	125 (76%)	0.68	113 (69%)	0.80	19 (12%)	0.87	30 (18%)	0.81	50 (30%)	0.87
Single (n = 253)	187 (74%)		170 (67%)		32 (13%)		50 (20%)		74 (29%)	
**Household Income**										
< R1000/m (n = 162)	118 (73%)	0.21	103 (64%)	0.17	22 (14%)	0.77	29 (18%)	0.61	41 (25%)	0.16
R1000-5000/m (n = 199)	156 (78%)		144 (72%)		22 (11%)		42 (21%)		68 (34%)	
>R5000/m (n = 56)	38 (68%)		36 (64%)		7 (12%)		9 (16%)		15 (27%)	

## Prevalence of Polyvictimisation 

Table 5 reports rates of polyvictimisation in the sample by each visit. By the age of 6 years, 49% of participants who were exposed to some form of violence, were exposed to multiple types of violence. Furthermore, sex differences were observed at the 4.5-year visit, where more boys (37%) experienced significantly more lifetime polyvictimisation than girls (31%). 

**Table 5.** Prevalence of Polyvictimization Stratified by Sex. *p-value*: Chi-square test.

Polyvictimisation	3.5 years ( *N* = 530)			4.5 years (N = 749)			6 years (N = 417)		
	Female (n = 262)	Male (n = 268)	*p-value*	Female (n = 371)	Male (n = 378)	*p-value*	Female (n = 198)	Male (n = 219)	*p-value*
Any Polyvictimisation	80 (31%)	99 (37%)	0.14	105 (28%)	145 (38%)	0.00	64 (32%)	91 (42%)	0.06
Number of different types of violence exposure									
None	82 (31%)	65 (24%)	0.32	98 (26%)	89 (24%)	0.01	48 (24%)	51 (23%)	0.25
One type	98 (37%)	104 (39%)		168 (45%)	143 (38%)		84 (42%)	72 (33%)	
Two types	52 (20%)	60 (22%)		75 (20%)	91 (24%)		42 (21%)	56 (26%)	
Three types	24 (9%)	32 (12%)		27 (7%)	41 (11%)		18 (9%)	29 (13%)	
Four types	6 (2%)	7 (3%)		3 (1%)	14 (4%)		6 (3%)	11 (5%)	

## Discussion

In this South African birth cohort, we found that a large proportion of children (72%-75%) were exposed to direct and indirect violent experiences in their homes as well as in the community from a very young age with substantial numbers experiencing polyvictimization. Boys and girls were similarly exposed to violence overall, but there was some evidence that boys were more vulnerable to *Domestic Victimisation* and polyvictimization by age 4.5 years of age.

By the age of 3.5 years, 72% of the children in this cohort had been exposed to some form of violence with *Witnessing Community Violence* as the most prevalent form of violence reported. This is consistent with the reports of high homicide rates and gang-related violence in the Western Cape region ( South African Police Service, 2021). Other studies with older children in the Western Cape also reported high exposure to community violence. One study found that 98.9% of their sample (aged 12–15 years) had witnessed community violence ( Kaminer *et al*., 2013c) whilst another found that 84.1% of their sample ( *Mean* age = 14.2 years) had been exposed to violence ( Stansfeld *et al*., 2017). A study providing national estimates of trauma exposure in South African adults also found high levels of witnessing violence ( Atwoli *et al*., 2013), suggesting that this form of trauma is potentially pervasive across the life span in some South African contexts.

Domestic violence was also commonly reported, consistent with earlier reports on IPV during pregnancy in this cohort ( Barnett *et al*., 2018). Similar prevalence estimates (20% – 31%) were reported on average for both witnessed domestic violent acts as well as violent acts directed at the child at home. Finding that reports of witnessed domestic violence decreased with age, unlike the other forms of violence, may however suggest underreporting of IPV. IPV and domestic violence are typically considered private matters and rely on people feeling able to divulge this sensitive information. Nonetheless, child exposure to IPV is limited, despite its occurrence; or many caregivers may be of the impression that their children are not being exposed to violence in the home, nor attending to or being affected by the incidences of IPV and domestic violence in the family. Furthermore, children’s social-emotional and cognitive development becomes more apparent to caregivers as the children grow older such that parents may appreciate the impact of domestic violence on the children and as such underreport children’s exposure to it. Additionally, it is possible that caregivers under-estimate the extent to which young children are aware of the violence occuring in parts of the home or after the child has gone to bed. Indeed preivous studies have found descripencies between parent and child reports of domestic violence exposure. Alternatively, it is also possible that children in our sample were witnessing less violence with age, given that younger children require more attention which places strain on caregiver relationships subsequently resulting in domestic violence. As such children may be at a greater risk of witnessing domestic violence at younger ages.

Although *Community Victimisation* is the least prevalent form of reported violence exposure in these early years, it is likely to increase in prevalence as the children get older and spend more time outside the home. Indeed other studies conducted in South Africa in older children found this type of violence highly prevalent in adolescents ( Haahr-Pedersen *et al*., 2020b; Kaminer *et al*., 2013e). The finding that a majority of children were exposed to community violence from as young as 3.5 years of age is particularly concerning, given associations between community violence exposure and later aggression ( Grasso *et al*., 2016), other mental health problems including posttraumatic stress disorder (PTSD) and internalising and externalizing behavioural problems in children and adolescents, respectively ( Fowler *et al*., 2009a).

Polyvictimisation was reported in this cohort, especially at the 6.5-year visit, where 49% of the children were reported to have been exposed to more than one type of violence, while at the 3.5- and 4.5-year visits the prevalence rates were 34% and 33% respectively. The higher prevalence at the later visit is possibly a result of older children being exposed to different settings beyond the family home such as school and the community where they may experience violence in these contexts in addition to that in the domestic environment. Indeed, even higher polyvictimisation prevalence has been reported in older children, for example, 93% of adolescents aged 12–15 years reported experiencing polyvictimisation in a Cape Town study ( Kaminer *et al*., 2013c) whilst another more recent national South African study reported polyvictimisation in 64% adolescents aged 15 – 17 years ( Leoschut & Kafaar, 2017). Finding high rates of polyvictimisation in our sample of such young children is important, given that research has shown that polyvictimisation may contribute to the experience of cumulative stress, aggravating later health outcomes and potentially altering developmental trajectories ( Appleyard *et al*., 2005; Teicher *et al*., 2006). Furthermore, polyvictimisation has been found to be a stronger risk factor for mental health problems than single forms of victimization (see review by ( Haahr-Pedersen *et al*., 2020c).

More boys than girls in this cohort were reportedly exposed to polyvictimisation by age 4.5 years. Studies have reported mixed findings regarding the relationship between sex and children’s exposure to violence. While our study used parent reports, other studies using self-report measures also found that older boys reported more exposure to violence than girls ( Falconer *et al*., 2021; Kamine *et al*., 2013c; Kaminer *et al*., 2013e; Shields *et al*., 2008) whilst others found no differences in reported exposure to violence ( Asagba *et al*., 2021; Finkelhor *et al*., 2015; Sternberg *et al*., 2006). Although research on sex differences in polyvictimization prevalence patterns is very limited, one other study also found that polyvictimization was higher in boys in their sample of adolescents aged 12–15 years living in Cape Town ( Kaminer *et al*., 2013c). Differential socialization of the sexes may explain this discrepancy as boys may be less protected than girls allowing them to spend more time outside the home, making them more vulnerable to other forms of violence. There was also some evidence in our study that boys experience more domestic victimisation, suggesting there could also be differences in parental perceptions of the suitability of harsh discipline practices for girls versus boys. Some studies have shown that boys are punished more frequently than girls due to differences in gender roles and expectations between the two sexes (see review by Lokot *et al*., 2020). Here, gender refers to the socially constructed roles, behaviours and identities of female, male and gender-diverse people( Coen & Banister, 2012).

South Africa’s political history of oppression and the structural and socioeconomic inequalities that have persisted in society are likely to contribute to the high levels of violence in these communities. These rates of violence exposure may not be generalisable to all South African communities. However, this cohort is representative of many communities in low and middle-income countries, with high rates of poverty, unemployment, and low levels of educational attainment among women. Given that the majority of studies reporting on children’s exposure to violence have mostly used samples of older children ( Falconer *et al*., 2021; Hillis *et al*., 2016; Hinsberger *et al*., 2016; Kaminer *et al*., 2013c; Kaminer *et al*., 2013e; Shields *et al*., 2008) finding that violence exposure is common in this sample of pre-schoolers suggests that many children experience the persistent threat of violence throughout the life course and importantly, during early formative years.

Finding no evidence of associations between violence exposure and maternal marital status or socioeconomic status in this cohort suggests that children in our study are similarly exposed to violence regardless of their background. This may be expected given that the DCHS children live in similar contexts where community violence is a common phenomenon. Furthermore, certain types of violence such as intimate partner violence and corporal punishment are widely accepted in the South African society ( Mathews *et al*., 2014). As such, children in our sample are exposed to similar rates of domestic violence regardless of their mother’s socioeconomic context.

Notably, violence exposure in early childhood occurs during sensitive and critical periods of development lasting from infancy to adolescence and can disrupt brain development and consequently impacting affective and neurocognitive systems ( Hertzman & Boyce, 2010) . Young children such as those in our sample experiencing violence during this period have increased risk of short term and long term poor developmental outcomes such as impaired socio-emotional development, mental health problems, poor cognitive functions and physical health problems in adulthood (see reviews: Fowler *et al*., 2009b; Maguire *et al*., 2015; Norman *et al*., 2012; Young-Southward *et al*., 2020). Previous research has highlighted the early childhood period as a key developmental stage where violence exposure occurring here is associated with greater health problems than violence occurring in other periods ( Cowell *et al*., 2015; Dunn *et al*., 2020; Vu *et al*., 2016). This is important given that existing literature investigating violence exposure has focused particularly on adolescents and adults, neglecting young children. As such, this, together with our findings, emphasize the need for closer attention to be paid to young children such as those in our sample, whose development is taking place in violent contexts. Notably, this descriptive study has provided context for future research to investigate the relatioships between violence exposure and socio-emotional, mental health and cognitve outcomes in young children in the DCHS and similar settings.

The findings of this study should be considered in view of some methodological limitations. Firstly, only a subsample of children was consistently seen at all three visits, such that the sample sizes differ at each visit and comprise different children. Secondly, there is no information about the exact timing of exposure to violence. Thirdly, given that violence exposure relied on caregiver reports and recall; exposure was likely underreported due to social desirability biases. This is a common limitation of studies that examine trauma exposure or maltreatment in children given that young children are unlikely to be developmentally capable of understanding the concept of a ‘violent act’ and reporting it adequately ( Shahinfar *et al*., 2000). Furthermore, obtaining reports of violence exposure from young children may risk re-traumatising them. The high prevalence rates of violence exposure reported are particularly important given the likelihood of underreporting in some domains. Fourthly, given that the 6-year visit had the smallest sample, the power to detect sex differences in polyvictimisation exposure patterns is limited. Lastly, we did not capture racial discrimination, another type of violence children in the DCHS communities may be experiencing given the persistent occurrence of racial discrimination in South Africa ( South African Human Rights Commission, 2021). Future research is needed to investigate the prevalence of this phenomenon in young South African children.

## Conclusion

These findings further highlight the high levels of violence exposure in South African children. They indicate that many pre-schoolers in our sample experience a pervasive threat of danger in their homes and communities. Interventions aimed at the community, family and individual levels, are crucial, not only to stop the cycle of violence but to help children deal with this trauma. Interventions aimed at building resilience in children may help them adapt psychologically. Over and above this, systematic factors that likely contribute to this picture such as poverty, socioeconomic inequality, high unemployment rates, low levels of educational attainment and weak police enforcement need to be mitigated to change this picture.

## Statements and declarations

1. The current analyses were funded by the Medical Research Council (MRC), grant ref. MR/T002816/1

2. The study was funded by the Bill and Melinda Gates Foundation (OPP 1017641). Additional funding was provided by the SA Medical Research Council, National Research Foundation, Academy of Medical Sciences Newton Advanced Fellowship (NAF002/1001) funded by the UK Government’s Newton Fund, by the National Institute on Alcohol Abuse and Alcoholism (NIAAA) via (R21AA023887, R01 AA026834-01), by a US Brain and Behaviour Foundation Independent Investigator grant (24467),

3. LT was supported by the University of Bristol’s (i) Pro Vice-Chancellor (PVC)-Research and Enterprise Strategic Research Fund and (ii) The Quality-related Research Global Challenges Research Fund (QRGCRF) Strategy funded by Research England.

## Patient consent

Parental/guardian consent was obtained.

## Ethics approval

The study protocol including consent forms was approved by the Faculty of Health Sciences Research Ethics Committee, University of Cape Town (401/2009), and by the Western Cape Provincial Research committee (2011RP45).

## Figures and Tables

**Supplementary Table 1.** Prevalence of Violence Exposure in Subsample.

	3.5 years	4.5 years	6. years
	N = 203		
Subscales			
Overall Violence Exposure	153 (75.4%)	148 (72.9%)	146 (71.9%)
Witnessing Community violence	132 (65.0%)	136 (67.0%)	134 (66.0%)
Community victimisation	12 (5.9%)	16 (7.9%)	26 (12.8%)
Witnessing Domestic Violence	56 (27.6%)	46 (22.7%)	36 (17.7%)
Domestic Victimisation	49 (24.1%)	45 (22.2%)	59 (29.1%)

Values indicate proportions of those with reports of violence exposure only

## Data Availability

Data are not publicly available. Collaborations for the analysis of data are welcome; the parent study has a large and active group of investigators and postgraduate students and many have successfully partnered with students or researchers from other institutions. Researchers who are interested in collaborations can find more information on our website [ http://www.paediatrics.uct.ac.za/scah/dclhs].

## Author’s information

LT is a Cotutelle PhD candidate at the University of Bristol and the University of Cape Town.
