# Supplementary material for: Early Childhood Violence Exposure Patterns in The Drakenstein Child Health Study (DCHS)
Source: Wellcome Open Res. 2023 Sep 19;8:36. Originally published 2023 Jan 24. [Version 2] doi: 10.12688/wellcomeopenres.18598.2 (PMC10534083; doi:10.12688/wellcomeopenres.18598.2)
Supplement: Peer review history

# Reviewer report: Reviewer response for version 2

**DOI:** 10.21956/wellcomeopenres.22162.r67293

**Published:** 2023-09-27

**Authors:** Kathy Sanders-Phillips

**Affiliations:** 1 Department of Pediatrics and Child Health, Howard University, Washington, District of Columbia, USA

I have reviewed the resubmission. The revisions are appropriate and sufficient to change my recommendation to *Approved.*

Is the work clearly and accurately presented and does it cite the current literature?

No

If applicable, is the statistical analysis and its interpretation appropriate?

No

Are all the source data underlying the results available to ensure full reproducibility?

Yes

Is the study design appropriate and is the work technically sound?

No

Are the conclusions drawn adequately supported by the results?

No

Are sufficient details of methods and analysis provided to allow replication by others?

Yes

Reviewer Expertise:

NA

I confirm that I have read this submission and believe that I have an appropriate level of expertise to confirm that it is of an acceptable scientific standard.

# Reviewer report: Reviewer response for version 1

**DOI:** 10.21956/wellcomeopenres.20623.r61608

**Published:** 2023-07-20

**Authors:** Helen Minnis

**Affiliations:** 1 School of Health and Wellbeing, Clarice Pears Building, University of Glasgow, Glasgow, UK

This is an impressive and important study which is, on the whole, clearly reported. In it, the team have recruited mothers mothers at 20-28 weeks gestation and followed up their children at 3.5, 4.5 and 6 years of age using a parent-report measure of the child’s lifetime exposure to domestic, school and community violence. As the authors state, there is a major international evidence gap regarding child development in the preschool years, since most longitudinal studies on “young people” focus on adolescents. Yet, as has been demonstrated by other authors, exposure to negative environments in the very early years can set a child on a trajectory towards violence (Waller *et al*, 2017 ^1^). Prospective longitudinal studies beginning in early childhood are rare in low to middle income contexts which makes the Drakenstein cohort particularly precious.

 Methods of recruitment were robust and well described, although small details could further improve reporting to demonstrate this even more clearly. For example, in the Figure 1 flow diagram, it would be helpful to include an additional box, between "approached” and “enrolled” for “eligible” and to include percentages, since this would show that the team were successful in recruiting nearly 70% of those eligible to take part and that, of the 980 active cases, they were able to assess 54% of the children at 3.5 years of age. For such a socially fragmented, high risk, community this is very impressive. The Results are generally very clear although, on page 5, (Results first paragraph) it would be helpful to make a clear statement that draws the reader’s attention to Table 1, which shows that the sample of 203 children with data at all three timepoints is highly representative of the full cohort. I also wonder whether the data in Tables 5 and 6 might be better displayed in histograms which would allow for easier comparison across gender and timepoints. It might also have been helpful to see the data in Table 6 presented by sex, although as the authors note the numbers would probably have been too small for inferential statistics to be useful.

 The Discussion is clear and compelling and the findings are important for public policy in South Africa and also internationally. The findings show that the history of colonialisation and apartheid has had a devastating impact on the Drakenstein community and, no doubt, similar findings would be found in many post-colonial LMIC across the world. The focus on polyvictimisation was important, especially in highlighting the burden of violence exposure and the gender differences. Even though a minority of young children in the cohort have experienced any one type of violence exposure, 72% have experienced violence exposure of some kind by age 6. Since previous research has shown that there seems to be a tipping point for greatly increased risk of developing aggressive or violent behaviours around age 2 years (Waller et al 2017 ^1^; King et al 2023 ^2^), a strong international focus on preventing violence exposure, especially in babies and young children, is warranted if this intergenerational pattern is to be broken. A strong early childhood health and social care focus is likely to be necessary if communities like Drakenstein are to recover from colonialisation and apartheid.

 Although the authors make important points about their findings clearly, and place them sensibly in the context of the international literature, there are one or two things it might be worth them considering in addition:

- In Table 2 and, as the authors point out in the Discussion (page 8, second para), there is an interesting opposite trend in which reports of children witnessing parents hitting one another are more common for younger children, compared to other forms of violence exposure which are more commonly reported for older children. The authors rightly suggest that social desirability might be the explanation. An alternative/additional explanation might be that caring for younger children is more challenging/stressful for parents leading to a higher risk of domestic violence, so that children might actually be at greater risk of witnessing domestic violence at younger ages.
- The authors suggest social desirability as an explanation for possible under-reporting of violence exposure in younger children – a very sensible point. An alternative/additional explanation might be the tendency of adults to under-estimate the degree to which young children have been aware of violence which occurred in other parts of the house, or after the child went to bed. Therapists working with young children with PTSD have sometimes found them to have witnessed more than parents and other family members had realised.  

Both of these additional points only make the international policy case for an early years focus on violence prevention even stronger.

 In summary, this is an impressive account of an important study and I look forward to seeing further publications in which the authors examine the associations between violence exposure and other variables as the children develop.

Is the work clearly and accurately presented and does it cite the current literature?

Yes

If applicable, is the statistical analysis and its interpretation appropriate?

Yes

Are all the source data underlying the results available to ensure full reproducibility?

Yes

Is the study design appropriate and is the work technically sound?

Yes

Are the conclusions drawn adequately supported by the results?

Yes

Are sufficient details of methods and analysis provided to allow replication by others?

Yes

Reviewer Expertise:

I am a child psychiatric epidemiologist and trialist with an interest in the psychopathology associated with abuse and neglect.

I confirm that I have read this submission and believe that I have an appropriate level of expertise to confirm that it is of an acceptable scientific standard.

## References

[1] : Observed fearlessness and positive parenting interact to predict childhood callous-unemotional behaviors among low-income boys. *J Child Psychol Psychiatry* .2017;58(3) : 10.1111/jcpp.12666 282-291 10.1111/jcpp.12666 27917472PMC5309210

[2] : A Comprehensive Multilevel Analysis of the Bucharest Early Intervention Project: Causal Effects on Recovery From Early Severe Deprivation. *Am J Psychiatry* .2023; 10.1176/appi.ajp.20220672 appiajp20220672 10.1176/appi.ajp.20220672 37211832PMC10751847

# Response

**Published:** 2023-09-03

**Authors:** lucinda tsunga

**Affiliations:** University of Bristol, South Africa

Thank you very much for your very helpful comments on our descriptive paper. We have update the manuscript as follows:

 Percentages have been added to the figure to highlight that 67% of the eligible participants were included.

 We have also included in the description of the sociodemographic data that the 203 children have similar sociodemographic characteristics to the rest of the cohort.

 We have added the suggested alternative explanations for the pattern of decrease in domestic violence exposure in the discussion. Indeed, histograms would have added another comparative element through visualisation, however we feel that the tables are better for our purposes of describing exposure patterns as frequencies are clearly presented in the tabular format.

# Reviewer report: Reviewer response for version 1

**DOI:** 10.21956/wellcomeopenres.20623.r56668

**Published:** 2023-06-22

**Authors:** Kathy Sanders-Phillips

**Affiliations:** 1 Department of Pediatrics and Child Health, Howard University, Washington, District of Columbia, USA

This manuscript describes a study of violence exposure among South African youth. The strengths of the study include its focus on youth aged 3 and 6 years and the collection of longitudinal data of both direct and indirect and domestic and community violence exposure as well as polyvictimization. However, the potential impact on the field is significantly limited by several factors. First, the participants were a non-random, voluntary sample. Nearly 20% of the individuals who were approached declined to participate. Thus, the original sample is potentially biased in an unknown direction.

 Second, only 203 of the original sample were evaluated at all three visits (3.5, 4.5, 6 years). This increases the possibility of sampling bias. The sampling bias may have also be fostered by administering the study instrument by in-person interviewers. As a result, social desirability may have influenced participant responses. In addition, since child violence exposure was assessed via caregiver report and recall, it is not clear whether child exposure or caregiver exposure was actually assessed in the study and/or whether caregiver exposure to domestic violence may influence the likelihood of accurate reporting.

 An additional concern is the failure in the study to fully address the potential role of racial discrimination as a form of violence exposure. For example, the article did not describe the racial/ethnic identity of the participants and the study did not assess historical and/or current racial discrimination. This is potentially problematic since existing data on polyvictimization indicates that exposure to racial discrimination among South African youth may exacerbate the impact of exposure to other types of violence. It is also possible that exposure to the violence of racism may influence caretaker perceptions of other types of violence exposures and increase the likelihood of violence in the home and community. Since the life experiences of individuals in South Africa were and continue to be significantly influenced by their racial categorization, it is not clear why perceived racial identification of participants was not identified in the manuscript. 

 Third, a more robust data analyses of the variables would have improved the study. Rather than chi square, for example, regression or profile analyses could have been utilized. As the manuscript notes, there may have been insufficient power to test some hypotheses in the sample at 6 years. There is also a lack of clarity on whether all the possible covariates such as illegal drug use were included in the analyses. 

 Fourth, there is little discussion of the impact of exposure to violence in the context of the development of 3-6 years olds. For example, the manuscript would have been improved had the authors speculated on the potential differences of first exposure at 3-6 versus first exposure at adolescence. This would have strengthened the potential importance of the findings for child development theory.

 In summary, given the number and significance of the limitations of this study, it is unlikely that the work will contribute to the growth of the field.

Is the work clearly and accurately presented and does it cite the current literature?

No

If applicable, is the statistical analysis and its interpretation appropriate?

No

Are all the source data underlying the results available to ensure full reproducibility?

Yes

Is the study design appropriate and is the work technically sound?

No

Are the conclusions drawn adequately supported by the results?

No

Are sufficient details of methods and analysis provided to allow replication by others?

Yes

Reviewer Expertise:

Child development, violence exposure, and racism in secondary social groups.

I confirm that I have read this submission and believe that I have an appropriate level of expertise to state that I do not consider it to be of an acceptable scientific standard, for reasons outlined above.

# Response

**Published:** 2023-09-03

**Authors:** lucinda tsunga

**Affiliations:** University of Bristol, South Africa

Thank you for your review and comments on our descriptive paper. Which we have addressed, and below we report detailed response to your concerns and outlined how we have amended our manuscript in light of this:

 Our study is a longitudinal prospective birth cohort, and as such, it aims to follow a group of individuals from birth throughout their lives to understand various aspects of health and development. The sample presented all participants who were available and eligible for enrolment during a particular time period thus randomization was not a consideration in our sample selection. Despite this limitation, we successfully recruited nearly 70% of those eligible to take part, which demonstrates a commendable effort in engaging and involving a substantial portion of the target population. In a socially fragmented and high-risk community, achieving such a participation rate is a significant accomplishment and adds value to the study's findings. We have updated the flow diagram to indicate percentages as a way of highlighting this. Furthermore, the study has shown low attrition rates with 980 children being active at the 6 year visit. While only a 749 children attended the 4.5 year CECV visit, similar attendance rates are reported at other study visits. Reasons for missing study visits include, caregivers temporarily moving out of the area and mother’s unavailability due to employment obligations.

 Regarding the issues of sampling bias you have highlighted: Table 1, shows that the sample of 203 children with data at all three timepoints is highly representative of the full cohort. We have now added this description in the text. Furthermore, supplementary table 1 shows how violence exposure patterns of the 203 are similar to the patters of the children reported in the main analysis.  

 We acknowledge that our violence exposure measure was conducted through in-person interviews, and this method may have introduced some level of bias. Participants' willingness to participate and provide responses could have been influenced by various factors, including social desirability. This method of gathering data is common in research contexts such as the one in our study with low literacy levels (see Sociodemographics).  

 As such, having an interview format allowed further clarification where needed. We now have included this explanation in our methods. Our research team took several measures to ensure the participants' comfort and reduce the impact of social desirability on responses. During the recruitment process, research assistants (psychology postgraduate students) were trained to be neutral, non-judgmental, and empathetic, creating a safe and non-threatening environment for the participants. We emphasized the voluntary and confidential nature of their participation to encourage honest and candid responses. This relationship has no doubt contributed to the low attrition rates in the study. We further discuss the impact of social desirability as a limitation but also highlight that despite this, the prevalence of violence in our sample is still significant.

 Regarding the clarity of who’s reporting on violence exposure and the likelihood of accurate reporting, as described under methods, the CECV questions asked caregivers about their child’s exposure to violence. More specifically, parents’ awareness of their children’s exposure. In the discussion, we highlighted the limitation that children’s exposure may be underreported due to social desirability. Furthermore, we discuss the issues surrounding children that age reporting on violence exposure. Indeed we did not investigate racial discrimination as a form of violence exposure, a limitation of the CECV. We have now added this as a limitation.

 Regarding our lack of disclosure of the racial (self-identified ancestry) composition of our sample, we made a decision not to focus on race in relation to violence patterns. Given the extensive history of race being used as a powerful dividing force in South Africa as part of the Apartheid Government, we did not want to perpetuate this practice. Instead, we focused on socioeconomic inequalities in relation to violence exposure patterns, with the majority of our sample belonging to a lower socioeconomic class given that evidence suggests higher levels of violence in contexts with high levels of socioeconomic inequality. South Africa being a good example of this. We have now added the racial composition of the sample merely as an additional descriptor of our sample. In this way we highlight the socioeconomic challenges that persist in the communities where our sample reside. These challenges are a product of the Apartheid Government which systematically segregated South African people on the basis of ancestry profoundly affecting various socioeconomic aspects of their lives. Given that the focus of the study was descriptive, chi-square analyses were deemed sufficient and do not require confounding control as such we did not look at many correlates. In our analyses we did look at three sociodemographic factors in relation to violence exposure patterns, namely, child sex, maternal marital status and household income. Evidence has shown previously how children can differ in terms of their violence exposure status based on these factors. While data on other factors is present in the DCHS, such analyses are beyond the scope of this paper. The intention of this paper was to detail the violence exposure in this cohort and the range of other environmental and psychosocial risks. We needed to give the exposure to violence in young children a particular focus because it has not been widely reported before. Furthermore, analyses of the violence exposure measure in this paper provided context for this data to be used in more integrated analysis in the future. Indeed, we did not discuss the impact of violence in 3-6 year old or in relations to adolescence. We have now added this aspect in the discussion.
